# Practical Recommendations for a Selection of Inhaled Corticosteroids in COPD: A Composite ICO Chart

**DOI:** 10.3390/biom13020213

**Published:** 2023-01-22

**Authors:** Keiji Oishi, Kazuto Matsunaga, Tasuku Yamamoto, Kazuki Matsuda, Yoriyuki Murata, Tsunahiko Hirano

**Affiliations:** Department of Respiratory Medicine and Infectious Disease, Graduate School of Medicine, Yamaguchi University, Ube 755-8505, Japan

**Keywords:** COPD, ICS, type 2 inflammatory biomarkers, asthma, exacerbations, pneumonia

## Abstract

The use of inhaled corticosteroids (ICS) for the maintenance of bronchodilator treatment in patients with chronic obstructive pulmonary disease (COPD) is controversial. While some patients achieve clinical benefits, such as fewer exacerbations and improved symptoms, others do not, and some experience undesired side effects, such as pneumonia. Thus, we reviewed the evidence related to predictors of ICS therapy treatment response in patients with COPD. The first priority clinical markers when considering the efficacy of ICS are type 2 inflammatory biomarkers, followed by a history of suspected asthma and recurrent exacerbations. It is also necessary to consider any potential infection risk associated with ICS, and several risk factors for pneumonia when using ICS have been clarified in recent years. In this article, based on the evidence supporting the selection of ICS for COPD, we propose an ICS composite that can be added to the COPD (ICO) chart for use in clinical practice. The chart divided the type 2 biomarkers into three ranges and provided recommendations (recommend, consider, and against) by combining the history of suspected asthma, history of exacerbations, and risk of infection.

## 1. Introduction

Chronic obstructive pulmonary disease (COPD) is a preventable and treatable disease; however, it presents a growing social and economic burden worldwide in terms of both disease prevalence and mortality [1]. The goals of COPD management include relieving symptoms, improving quality of life (QOL), maintaining or improving exercise tolerance and physical activity, preventing exacerbations and disease progression, and reducing premature mortality [2,3]. Both pharmacologic therapies and nonpharmacologic treatments, such as smoking cessation and pulmonary rehabilitation, are important in achieving management goals. Bronchodilator therapy with long-acting muscarinic antagonists (LAMA), long-acting beta-agonists (LABA), or combinations of both, is considered by the various guidelines as the main pharmacotherapy of COPD [2,3,4,5,6]. Long-acting bronchodilators can reduce exacerbation and improve lung function, exercise capacity, symptoms, and QOL in patients with COPD [7]. On the other hand, many patients have residual symptoms and repeated exacerbations despite optimal bronchodilator therapy.

The addition of Inhaled corticosteroids (ICS) to regular bronchodilator treatment in patients with COPD has been debated back and forth for a long time. ICS recommendations have changed over time in the Global Initiative for Chronic Obstructive Lung Disease (GOLD) reports and guidelines for different countries. The GOLD 2023 report recommends the use of ICS as step-up pharmacologic therapy for COPD patients with frequent exacerbations despite regular treatment with bronchodilators and evidence of eosinophilic inflammation (blood eosinophil count of >300 cells/µL) [2]. Treatment with ICS added to bronchodilators has been reported to reduce exacerbations and improve symptoms in patients with uncontrolled COPD [8,9,10,11,12,13,14]. For COPD patients who have frequent exacerbations even with dual bronchodilators, recent studies have demonstrated that the addition of ICS not only improves exacerbations and symptoms but also reduces death [15,16]. Subgroup analyses of many clinical trials have shown that type 2 inflammation and concomitant asthma are useful indicators to predict ICS-treatment response [15,17,18]. On the other hand, there is concern about the risk of increased respiratory infections as a side effect of ICS. In recent years, several characteristics were clarified for risk factors for pneumonia when using ICS [19,20,21]. Despite the GOLD recommendations, evidence from real-world studies suggests that ICS is being over-prescribed in COPD, irrespective of disease presentation and underlying inflammation [22,23,24].

Therefore, a combined evaluation of patient-specific predictors of response and risk factors, such as (1) type 2 inflammatory biomarkers, (2) history of suspected asthma, (3) history of exacerbations, and (4) risk of infection, is required to select ICS more safely and effectively in the management of COPD. In this review, we propose a composite Ics in COpd (ICO) chart that can be practically applied, based on the evidence for ICS selection for COPD.

## 2. Type 2 Inflammation Biomarker

### 2.1. Type 2 Inflammatory Biomarker in COPD

COPD has heterogeneous patterns in the inflammatory process. Representative inflammatory cells of the airway in COPD are neutrophils, which reflect type 1 inflammation. There are also phenotypes of COPD in which eosinophilic inflammation of the airway is predominant under circumstances such as exacerbation or asthma overlap. In short, eosinophilic inflammation in the disease is a promising therapeutic target because sputum eosinophilia becomes a predictor of clinical outcomes [25]. Eosinophilic inflammation in the airway is thought to reflect type 2 inflammation caused by T helper 2 (Th2) lymphocytes from adaptive immune systems (allergic eosinophilic airway inflammation) and innate lymphoid group 2 cells (ILC2) from innate systems (non-allergic eosinophilic airway inflammation) [26]. However, it is not easy for clinicians to measure the inflammatory status of the airways by using a sputum examination. Instead, in a clinical setting, type 2 inflammatory biomarkers, such as blood eosinophil and fractional exhaled nitric oxide (FeNO), are measured as surrogate markers of eosinophilic inflammation of the airway because these biomarkers are easily accessible and useful indicators to predict the exacerbation risk and treatment response [27,28,29].

### 2.2. Relationship between Type 2 Biomarkers and Clinical Outcomes of COPD

Adaptive or innate immune system dysregulation overproduces type 2 cytokines, such as interleukin (IL)-5, IL-4/13, granulocyte-macrophage colony-stimulating factor (GM-CSF), IL-33, and thymic stromal lymphopoietin (TSLP) [30,31]. As a result, the elevation of type 2 biomarkers, such as eosinophils and FeNO, persist. Both type 2 biomarkers in COPD have actually been reported to become predictors for symptom burden, pulmonary function decline, and exacerbation risk [32]. Moreover, they have increasingly been highlighted by recent evidence because they seem to be very promising tools to identify which patients with COPD are most likely to benefit from ICS [27]. Hereafter, the relationship between ICS response to clinical outcomes and blood eosinophils and FeNO is described.

#### 2.2.1. ICS Effect on Symptom Burden

**Figure 1:**
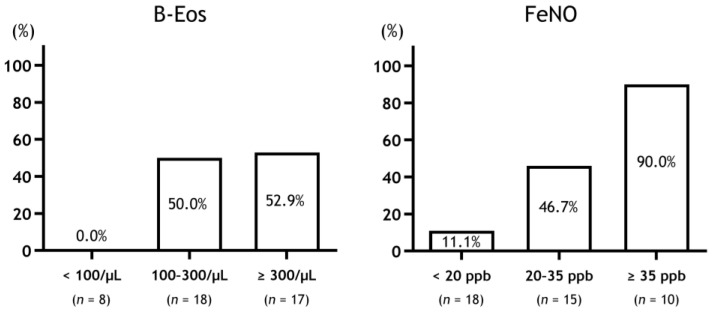
Response rate to ICS therapy in patients with COPD stratified by type 2 biomarkers. Abbreviations: B-Eos, blood eosinophil counts; FeNO, fractional exhaled nitric oxide.

Although ICS at higher blood eosinophil counts (≥310 cells/μL) is effective for symptom relief in COPD, lower blood eosinophil counts (<90 cells/μL) are not effective [17]. Additionally, high FeNO levels (≥25 ppb) in patients with COPD could become better predictors for the ICS/LABA effect on symptomatic relief compared to patients with low FeNO levels [33]. In the Destress study, while the patients with COPD with FeNO > 35 ppb had improved symptoms in response to ICS, those with FeNO < 20 ppb did not improve [29]. Moreover, when the patients with COPD were divided into three groups according to FeNO levels, the low group (<25 ppb) had few responders, while the intermediate (20–35 ppb) and high groups (≥35 ppb) had more responders in that order. This was also the case when the patients with COPD were divided into three groups according to blood eosinophil counts (Figure 1).

#### 2.2.2. ICS Effect on Pulmonary Function Decline

Patients with COPD with higher blood eosinophil counts (≥220 cells/μL) showed a stronger bronchodilator effect of ICS compared to those with lower blood eosinophil counts. In particular, patients with COPD with blood eosinophil counts >270 cells/μL showed clinically important treatment differences in lung function (FEV_1_ ≥ 50 mL) [18]. In addition, ICS users with COPD with higher blood eosinophil levels (≥2%) showed a slower FEV_1_ decline [34]. Moreover, Kerkhof et al. showed that patients with COPD with high blood eosinophil counts (≥350 cells/μL) and at least one instance of exacerbation had a significantly greater FEV_1_ decline if they were not treated with ICS. This suggests that ICS is an important strategy for preventing the rapid loss of lung function, which is caused by eosinophilic exacerbations in patients with COPD [35]. In contrast, a higher FeNO value (>35 ppb) is a good predictor of increased pulmonary function (FEV_1_) by ICS, while poor bronchodilator responsiveness after ICS use is predictable by a lower FeNO value (<20 ppb) [28,29].

#### 2.2.3. ICS Effect on Exacerbation Risk

ICS is possibly beneficial for patients with COPD with elevated blood eosinophil counts (>150 cells/μL) to reduce exacerbations [15]. During severe exacerbations of COPD, ICS effectiveness is associated with the absolute number of blood eosinophils (≥200 cells/μL) [36]. At a low eosinophil count (<90 cells/μL), the moderate/severe exacerbation risk in once-daily single-inhaler triple therapy (SITT)(ICS/LABA/LAMA) was not reduced compared with that in LAMA/LABA (95% confidence interval: 0.88 [0.74, 1.04]), while the exacerbation rate ratio for triple therapy was significantly suppressed at high blood eosinophil counts (≥290 cells/μL) [17]. Patients with COPD with blood eosinophil counts >150 cells/μL are more likely to benefit, in terms of exacerbation risk, from triple therapy [15]. Therefore, high blood eosinophil levels (>300 cells/μL) can be a predictor for the exacerbation risk or better response to ICS in COPD [15,18,37,38,39,40,41]. Based on the aforementioned recent pieces of evidence, GOLD also recommends thresholds of blood eosinophils as a guide for ICS treatment in patients with COPD according to the exacerbation pattern [2]. Eosinophil counts ≥100 cells/μL accompanied with high exacerbation risk despite treatment with LAMA/LABA actually seem to have a useful index of proper use of triple therapy, including ICS [42]. However, blood eosinophil counts <100 cells/μL are not recommended for patients with COPD. Persistently high FeNO levels (≥20 ppb) seem to be a valuable indicator of an acute exacerbation in patients with stable COPD [43]. However, it remains uncertain whether FeNO could be a biomarker used to detect ICS responders for exacerbation risk.

#### 2.2.4. ICS Effect on Mortality

The benefits for COPD mortality with type 2 biomarker-targeted ICS treatment remain unclear. Only one study to date examined this point. The ETHOS Trial showed that the benefit of ICS/LABA/LAMA versus LABA/LAMA in reducing mortality generally increased with blood eosinophil count [44]. Future studies will be necessary to clarify this issue.

#### 2.2.5. Future Directions

Future directions include novel type 2 biomarkers and genetic studies. Emerging type 2 biomarkers, such as eosinophil cationic protein and eosinophil-derived neurotoxin (EDN), might be useful for identifying ICS treatment in patients with COPD. Although there are no reports on the effectiveness of these biomarkers in discriminating ICS treatment in COPD patients, a previous study reported that EDN was significantly higher in asthma–COPD overlap (ACO) than in asthma or COPD [32]. Several genome-wide association studies in COPD patients investigated the ICS potential genetic predictors of interindividual responses to ICS. In Chinese COPD patients, the single nucleotide polymorphisms (SNP) rs37973 may be linked to decreased ICS efficacy [45]. Another study revealed that the SNP rs111720447 was associated with lung function decline in COPD patients receiving ICS [46]. Although clinical implementation remains far away, these novel biomarkers and genetic studies are an area of great promise.

### 2.3. Brief Summary

The blood eosinophil count and FeNO could become surrogate markers for eosinophilic airway inflammation and are easily accessible in COPD. Moreover, they can predict the ICS response to symptom burden, pulmonary function decline, exacerbation risks, and death in COPD. These biomarkers could be a useful indicator to identify which patients with COPD would most likely benefit from ICS. Therefore, type 2 biomarker-targeted ICS therapy could contribute to the progress of medical efficiency in COPD.

## 3. History of Suspected Asthma

ICS is considered as the mainstay of treatment for patients with asthma [47]. However, no studies have examined the response to ICS in patients having COPD with a history of suspected asthma. Some reports have studied the proportion of patients having COPD with a history of suspected asthma and the association between medical history and type 2 inflammation. The coexistence rate of asthma in patients with COPD varies according to the definition and population. However, a previous systematic review reported a coexistence rate of 27% [48]. In this review, most of the articles included the history of asthma in the definition of asthma-related complications. Annangi et al. studied approximately 3.11 million Americans with COPD who were aged ≥40 years. They found that 14.6% of these patients had a history of asthma [49]. They also reported that 35.8% of the patients having COPD with a history of asthma had elevated blood eosinophil counts (≥300 cells/μL), and 84.4% had elevated FeNO levels (≥25 ppb). Thus, many patients having COPD with a history of asthma are believed to have type 2 airway inflammation. In contrast, in the present report, approximately 35.6% of the patients having COPD without a history of asthma had elevated blood eosinophil counts, and 15.2% had elevated FeNO levels. In other words, not all patients having COPD with type 2 airway inflammation had a history of asthma. Thus, this study suggests that a history of previously diagnosed asthma is an important finding that indicates a complication of asthma with type 2 airway inflammation.

The Japanese Respiratory Society published diagnostic criteria for ACO in 2018 [50]. These criteria use subjective information, such as variability of symptoms and a history of asthma before the age of 40 years, as well as objective information, such as FeNO level, blood eosinophil count, and airway reversibility, to arrive at a diagnosis. These criteria are used to distinguish between the pathophysiologies of asthma and COPD while making a diagnosis of ACO. In a multicenter prospective cohort study of approximately 400 patients, the prevalence of ACO among patients with COPD was reported to be 25.5% based on these criteria [51]. In this study, 27.3% of the patients with ACO had a history of asthma before the age of 40, and 85.7% of the patients were reported to have variable or paroxysmal respiratory symptoms. In addition, 68.6% of the patients with ACO had elevated FeNO levels (≥35 ppb), and 76.6% of the patients had allergic rhinitis or airway reversibility, elevated blood eosinophil counts (≥300 cells/µL), or high IgE levels. Type 2 inflammation is likely to be present in many patients with ACO who meet these diagnostic criteria. Therefore, a history of suspected asthma is thought to be a predictive biomarker of type 2 airway inflammation, which may be expected to respond to ICS.

In this section, we have described how variable or paroxysmal respiratory symptoms and a history of asthma before the age of 40 years are associated with the presence of type 2 inflammation. As discussed in a previous section, the presence of type 2 inflammation is associated with responsiveness to ICS in patients with COPD. Therefore, a history of suspected asthma constitutes a useful guide for the use of ICS in combination with the evaluation of blood eosinophil count and FeNO.

## 4. History of Exacerbations

### 4.1. Importance of Reducing COPD Exacerbations

Exacerbations in COPD are defined as an acute worsening of a patient’s condition, which includes respiratory symptoms and necessitates a change in regular medication [52,53]. Exacerbation is associated with a decline in lung function [54,55], quality of life [56], and poor prognosis in COPD patients [57]. Previous exacerbation in the past 12 months was the strongest risk factor for further exacerbation in COPD patients (odds ratio, 4.30; 95% confidence interval [CI], 3.58 to 5.17) [58]. This result has also been reported in many other studies [59]. Similarly, previous exacerbations increase subsequent severe exacerbations and mortality [60]. A recent study revealed that 36% of patients with no exacerbations at baseline will experience an exacerbation within the next three years [61]. Moreover, the importance of a single exacerbation is highlighted by the fact that even one moderate or severe exacerbation is a significant risk factor for all-cause mortality and re-exacerbation [62]. Therefore, it is important to reduce COPD exacerbation and keep exacerbation at zero. ICSs are expected to be useful for this purpose.

### 4.2. Usefulness of ICS in Reducing Exacerbations

In 2000, the ISOLDE trial revealed that the exacerbation rate of COPD was reduced by ICS (fluticasone propionate) compared to placebo [63]. After this trial, similar results have been reported, suggesting that ICS significantly suppresses exacerbation in some COPD patients with or without an exacerbation history [64,65,66]. Furthermore, ICS has been found to be more reliable when used in combination with a long-activating β agonist (LABA) compared with LABA alone for COPD patients with a history of at least one previous exacerbation [8,9,10,11,12,13,14,67]. Therefore, some have recommended that ICS be added to LABA for COPD patients with a high exacerbation risk, such as those with frequent exacerbations [68].

Conversely, long-activating muscarinic antagonists (LAMA) have also been reported to reduce exacerbation in COPD patients regardless of exacerbation history [69], and those with at least one exacerbation history per year [70]. However, the INSPIRE study showed no difference in the frequency of exacerbations between LAMA and ICS/LABA in COPD patients with an exacerbation history [71]. These findings suggest that if LAMA is used to treat COPD, ICS may not be necessary to prevent subsequent exacerbations.

Moreover, the WISDOM study showed that withdrawal of ICS did not increase exacerbation risk (hazard ratio, 1.06; 95% CI, 0.94 to 1.19) in COPD patients who received triple therapy (defined as treatment with LAMA, LABA, and ICS), with a history of at least one exacerbation in the past year [72]. Similar results were shown in moderate-to-severe COPD patients with no exacerbation history [73] and low exacerbation risk (FEV1 >50% of predicted and less than two exacerbations in the past year) [74]. In contrast, LAMA/LABA was reported to be more effective at preventing exacerbations than ICS/LABA in COPD patients with a history of at least one exacerbation in the previous year (the FLAME study) [75], and a meta-analysis that included FLAME and other studies showed the same result (LAMA/LABA vs. ICS/LABA, hazard ratio 0.82; 95% CI, 0.70 to 0.96) [76]. However, the WISDOM study showed that both a high blood eosinophil count (≥300 cells/μL) [40] and frequent exacerbation (two or more per year) are risk factors for exacerbation when ICS is withdrawn [41]. Thus, ICS should be considered when asthma complicates and/or exacerbates its frequency (two or more times per year).

### 4.3. Benefits and Problems of Triple Therapy with LAMA, LABA, and ICS

The effect of triple therapy with a single inhaler has recently been reported. First, the TRIBUTE trial showed that a single-inhaler triple therapy with beclomethasone/formoterol/glycopyrronium) decreased moderate-to-severe exacerbation compared with LAMA/LABA (glycopyrronium/indacaterol) (rate ratio, 0.848; 95% CI, 0.723–0.995) in COPD patients with FEV1 < 50% of predicted, moderate or severe exacerbation history, and without current asthma [77]. Second, the IMPACT trial, a large study, compared triple therapy with a single inhaler, LAMA/LABA, and ICS/LABA using the same ICS (fluticasone furoate), LABA (vilanterol), and LAMA (umeclidinium). This trial included COPD patients with FEV1 < 50% of the predicted value and a history of at least one moderate or severe exacerbation in the previous year, but those with asthma were explicitly excluded. Triple therapy resulted in a lower exacerbation rate than LAMA/LABA (rate ratio 0.75; 95% CI, 0.70 to 0.81) or ICS/LABA (rate ratio 0.85; 95% CI, 0.80 to 0.90) [16]. In addition to these studies, the ETHOS trial comparing triple therapy with LAMA/LABA or ICS/LABA in COPD patients with exacerbation history also showed similar results to triple therapy, with a greater ability to reduce exacerbation than LAMA/LABA (rate ratio, 0.76; 95% CI, 0.69 to 0.83), using the same ICS (budesonide), LABA (formoterol), and LAMA (glycopyrrolate) [15].

However, some problems have been pointed out in these studies showing the effects of triple therapy [78]. These studies excluded patients with current asthma but allowed past asthma. The population of patients who had been using ICS before study entry was approximately 60% in TRIBUTE, 70% in IMPACT, and 80% in ETHOS. Therefore, it has been pointed out that some of the LAMA/LABA groups may have increased the frequency of exacerbation because ICS, a necessary therapy for COPD with asthma-like features, was discontinued for study entry. Another article also reported that blood eosinophil counts are associated with reducing exacerbation rate in the IMPACT trial [17].

Conversely, in the subgroup analysis of the IMPACT study, the previous single moderate exacerbation group did not show a significant difference between triple therapy and LAMA (rate ratio, 0.92; 95% CI, 0.79 to 1.06); however, the frequent moderate exacerbation group and severe exacerbation group, which included patients who required hospitalization, showed significant effects (frequent group, rate ratio, 0.80; 95% CI, 0.72 to 0.90) (severe group, rate ratio, 0.81; 95% CI, 0.70 to 0.93) [79]. Additionally, in real-world clinical practice, Suissa et al. showed that the superiority of triple therapy over LAMA/LABA in preventing COPD exacerbation is exhibited in the blood eosinophil count >6% (hazard ratio, 0.83; 95% CI, 0.46 to 0.94) or frequent exacerbation (hazard ratio, 0.83; 95% CI, 0.70 to 0.98) [80]. The same investigators also recently reported that triple therapy has a higher mortality rate than LAMA/LABA in patients with no prior asthma diagnosis or none/one exacerbation in the previous year, but not prior asthma diagnosis and two or more exacerbations [81].

In summary, exacerbation history during the previous year is considered to not provide sufficient evidence for adding ICS to LAMA/LABA in patients with COPD. However, when blood eosinophils are high and frequent exacerbations occur, there is evidence for adding ICS to prevent further exacerbation. For patients with COPD and a history of frequent exacerbation but <300 cells/μL, it is controversial due to a lack of evidence.

## 5. Risk of Infection

### 5.1. Risk Factors of Respiratory Infections with ICS Treatment for Patients with COPD

While there are benefits that ICS bring to patients with COPD, such as a reduction of the frequency of exacerbations mainly caused by infectious mechanism, some concerns have been reported, especially, paradoxically, the increased risk of other respiratory infections. Infection-induced exacerbation of COPD significantly reduces patients’ prognosis and quality of life. Older age, lower body mass index (BMI), more severe airflow limitations, and use of high-dose ICS are generally associated with an increased risk of developing (and exacerbating) respiratory tract infections and pneumonia. Given these concerns, it is important to stratify risk by the patient and make individual and judicious decisions for ICS indications. According to the previous study [82], ICS was associated with a dose-dependent increased risk of acquiring *Haemophilus influenzae* (*H. influenzae*), and the authors said that high-dose ICS should be used with caution. *H. influenzae* is known to contribute to daily symptoms, exacerbations, and disease progression. Patients from whom *H. influenzae* was isolated also had lower BMI, lower FEV1, and more hospitalizations for previous exacerbations. Similarly, ICS dose-dependently increases the risk of *Pseudomonas aeruginosa* (*P. aeruginosa*) colonization [83]. *P. aeruginosa* was also more prevalent in patients with lower BMI, lower FEV1, and a higher rate of previously hospitalized exacerbations. It is also pointed out that ICS is related to non-tuberculous mycobacteriosis (NTM). Patients with COPD on current ICS therapy had about four times the odds ratios for the risk of NTM compared to patients with COPD who had never received ICS. The risk of ICS for NTM was dose-dependent, and fluticasone had a higher odds ratio (OR) than budesonide [84,85]. Giorgio Castellana et al. asserted the tuberculosis risk of ICS in a meta-analysis of non-randomized studies [86].

The mechanism by which these types of bacteria colonize is unclear; however, it is said that ICS can alternate the innate and adaptive immune system, increase the bacterial load and change the microbial composition in the airway, especially in patients with lower sputum or blood eosinophil [87]. Further possible mechanisms include easy infection with viruses and chronic respiratory tract infection due to suppression of the production of type-1 IFN and cathelicidin, an antimicrobial peptide [88]. ICS can be also involved in the deficiency of mucosal-associated invariant T (MAIT) cells. MAIT cells are a subset of innate-like T lymphocytes accounting for up to 10% of T cells in blood and airway tissue and play an important role in protective immunity against bacterial or fungal infections [89,90].

### 5.2. How Do We Measure the Risk of ICS for Infections in Patients with COPD?

Chronic bronchiectasis infection (CBI) (three or more times detections of the same causative bacterium in four consecutive valid sputum samples) in COPD patients is associated with the risk of developing pneumonia regardless of the number of blood eosinophils (100 or more or less than 100). Blood eosinophils count <100 cells/μL was the sole risk factor for pneumonia with or without CBI. ICS also increased the risk of developing pneumonia in patients with CBI and blood eosinophils count <100 cells/μL. On the other hand, the use of ICS in patients lacking these risk factors did not significantly increase the incidence of pneumonia [21]. It means that regular sputum cultures, reference to past sputum culture results, and confirmation by peripheral blood tests are important. Analysis of the TORCH study on pneumonia risk in COPD patients receiving ICS, the risk of developing pneumonia is associated with advanced age (≥55 years old), %FEV1 < 50% (namely GOLD stage III or higher), exacerbation within one year, and lower BMI (BMI < 25) [19]. Gender differences were not detected in this study. Moreover, Courtney Crim et al. clarified that current smoking, previous history of pneumonia (within one year), lower BMI (BMI < 25), and more severe airflow limitation (%FEV1 < 50%) are more than double the risk of developing pneumonia in ICS administration (fluticasone furoate + vilanterol vs. vilanterol alone) [20]. A tendency of dose-dependent risk was observed, especially in male patients.

Based on the above, in general, elderly patients (especially males) with lower BMI and more severe airflow limitation (GOLD III or higher) have a high risk of respiratory tract infection (including CBI, NTM, and pneumonia) and exacerbation due to the use of ICS; therefore, careful use of ICS should be considered. Histories of recurrent hospitalizations for exacerbations may reflect these risks. Consider ICS indications in patients with blood eosinophils count >100 cells/μL and no continuous detection of pathogenic bacteria in past sputum tests. However, the risk of ICS for infection is thought to increase in a dose-dependent manner; therefore, aimlessly continuous administration of high doses should be avoided.

## 6. Discussion

**Figure 2:**
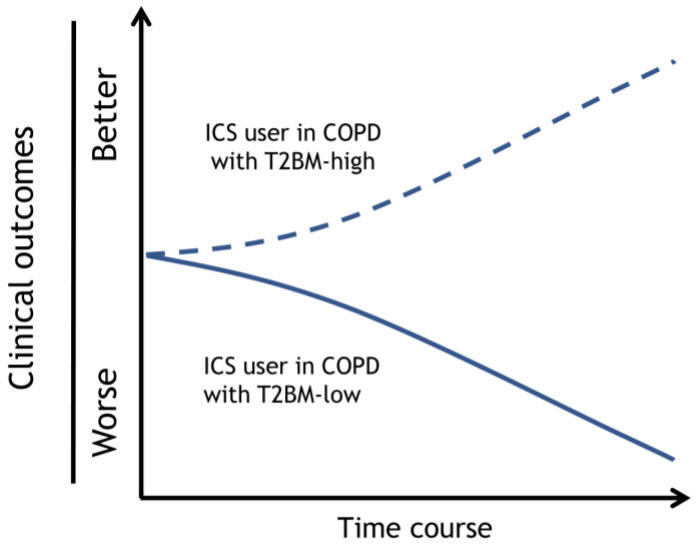
Prognosis of an ICS user with COPD with type 2 biomarker-high or -low. The clinical outcomes of the ICS user in COPD with T2BM-high can be improved, and T2BM-low exacerbated. Dotted and solid lines depict the time course of clinical outcomes, such as symptoms, pulmonary function decline, and exacerbations, by each strategy. Dotted lines; ICS user with COPD with T2BM-high, Solid lines; ICS user with COPD with T2BM-low. Adapted with permission from Ref. [27]. Copyright 2023 Japanese Society of Internal Medicine. Abbreviations: ICS, inhaled corticosteroid; COPD, chronic obstructive pulmonary disease; T2BM, type 2 biomarker.

According to the evidence discussed above, some patients with COPD may benefit from the addition of ICS to their bronchodilator treatment, while others may not. As a result, each patient’s risk/benefit ratio for starting ICS therapy must be carefully considered. Notably, the challenge is to determine what characteristics can be practically used to help identify patients with COPD who can benefit most from using ICS while running the lowest risk of unfavorable side effects. Based on a review of the current literature, type 2 inflammation biomarkers should be considered the highest priority as clinical markers of potential ICS benefits, such as shown in Figure 2. The next highest priority was a history of suspected asthma, followed by a history of COPD exacerbation. Furthermore, it is necessary to consider the potential risk of ICS infections. Therefore, we propose the following composite ICO chart to be considered when adding ICS treatment in combination with one or two long-acting bronchodilators (Table 1). The chart divided the type 2 inflammation biomarkers into three ranges and offered recommendations (recommend, consider, and against) by combining the history of suspected asthma, history of exacerbations, and risk of infection.

For the type 2 biomarker-high groups, such as patients with a blood eosinophil count of ≥300 cells/µL and/or FeNO ≥ 35 ppb, the current evidence is sufficient to make a firm recommendation regarding the use of ICS if there is a history of suspected asthma complications and/or frequent COPD exacerbations [15,17,18,41,44,78,80]. However, if such patients also have characteristics that place them at a high risk of infection, careful follow-up is needed to check for pneumonia development after ICS use. On the other hand, recommendations for the use of ICS are slightly lower for patients with type 2 biomarkers who do not have a history of suspected asthma complications or frequent COPD exacerbations and are more pronounced when the patient is at risk for infection [28,29,81].

For the type 2 biomarker-low groups, such as patients with a blood eosinophil count of <100 cells/µL and/or FeNO < 20 ppb, the use of ICS should normally be avoided [15,17,28,29,44]. However, it may be considered when there is a history of suspected asthma complications and little concern about infection.

For the type 2 biomarker-intermediate groups, such as patients with a blood eosinophil count of 100–300 cells/µL and/or FeNO 20–35 ppb, the current evidence is insufficient to make a firm recommendation. This is because most analyses are conducted for low and high type 2 biomarker groups, and few analyses focus on intermediate groups. For example, a post-hoc analysis of the KRONOS study is described below [91]. This analysis evaluated lung function and exacerbations in patients with moderate-to-very severe COPD who did not have airway reversibility and had a blood eosinophil count  of <300 cells/µL. The results showed that triple therapy did not significantly improve through FEV1 compared with LABA/LAMA, but significantly reduced the rate of moderate-to-severe exacerbations. While these findings are important, it was unclear how many patients with blood eosinophil counts below 100 were included; consequently, the results of the analysis in the 100–300 cells/µL population remain ambiguous. However, the results of the subgroup analyses of the IMPACT and ETHOS studies showed that even in the range of blood eosinophil counts from 100 to 300, the exacerbation–suppression effect of the addition of ICS gradually increased [15,17]. Moreover, the analysis in the Destress study showed no ICS responders in the low-type 2 biomarker group, while ICS responders were present in the intermediate group. Based on these results, we determined that the ICS recommendations could be expanded in the intermediate group compared to the low group and, thus, decided on the recommendation level.

A limitation of the ICO chart is that it does not provide recommendations when multiple factors are in conflict. The more multiple indicators a person has, the more difficult it becomes to combine and interpret them. In most patients, combinations of two or three are limited. However, each patient often must clarify which specific medical history must be prioritized. In the AERIS study, the analysis of COPD exacerbation phenotypes using a Markov chain model was utilized to show that bacterial and eosinophilic exacerbations were more likely to be repeated in subsequent exacerbations within a patient [92]. An analysis and a recommendation chart that combines all of the effects and risk factors presented here would be ideal, but the solution to such a request would require an Artificial Intelligence-based analysis that includes multiple factors. Thus, further validation and revision studies of ICO charts are required.

## Figures and Tables

**Table 1:** Composite ICO chart.

Blood eosinophils counts (BEC)	<100 cells/µL	100–300 cells/µL	≥300 cells/µL
Fractional exhaled nitric oxide (FeNO)	<20 ppb	20–35 ppb	≥35 ppb
History of suspected asthma	△	◯	◯
History of COPD exacerbation	×	△	◯
Risk of infection *	×	×	△
◯: Recommend ICS use △: Consider ICS use ×: Against ICS use			

* Carefully monitor for infectious concerns even when using ICS on the basis of a history of suspected asthma and/or of COPD exacerbation. Consider the discontinuation or dose reduction of ICS in cases of recurrent respiratory infections. Abbreviations: ICS, inhaled corticosteroid; COPD, chronic obstructive pulmonary disease.
